# Skullcap (*Scutellaria baicalensis*) Extract and Its Active Compound, Wogonin, Inhibit Ovalbumin-Induced Th2-Mediated Response

**DOI:** 10.3390/molecules19022536

**Published:** 2014-02-21

**Authors:** Hee Soon Shin, Min-Jung Bae, Dae Woon Choi, Dong-Hwa Shon

**Affiliations:** 1 Division of Metabolism & Functionality Research, Korea Food Research Institute, 1201-62, Anyangpangyo-ro, Bundang-gu, Seongnam-si, Gyeonggi-do 463-746, Korea; E-Mails: hsshin@kfri.re.kr (H.S.S.); mjbae1231@snu.ac.kr (M.-J.B.); choidw19@gmail.com (D.W.C.); 2 Institute for Basic Science, School of Biological Sciences, Seoul National University, 599, Gwanak-ro, Gwanak-gu, Seoul 151-742, Korea

**Keywords:** skullcap, wogonin, anti-allergy, IgE, IL-5, Th2 response

## Abstract

Skullcap *(Scutellaria baicalensis*) has been widely used as a dietary ingredient and traditional herbal medicine owing to its anti-inflammatory and anticancer properties. In this study, we investigated the anti-allergic effects of skullcap and its active compounds, focusing on T cell-mediated responses* ex vivo* and* in vivo*. Splenocytes from mice sensitized with ovalbumin (OVA) were isolated for analyses of cytokine production and cell viability. Mice sensitized with OVA were orally administered skullcap or wogonin for 16 days, and then immunoglobulin (Ig) and cytokine levels were measured by enzyme-linked immunosorbent assays. Treatment with skullcap significantly inhibited interleukin (IL)-4 production without reduction of cell viability. Moreover, wogonin, but not baicalin and baicalein, suppressed IL-4 and interferon-gamma production. *In vivo*, skullcap and wogonin downregulated OVA-induced Th2 immune responses, especially IgE and IL-5 prediction. Wogonin as an active component of skullcap may be applied as a therapeutic agent for IgE- and IL-5-mediated allergic disorders.

## 1. Introduction

Skullcap *(Scutellaria baicalensis*) is widely used as both a dietary ingredient and as a traditional herbal medicine in China, Japan, and Korea, to treat inflammation, allergy, and bacterial and viral infections [1,2]. For example, skullcap suppresses 48/80-induced histamine release in rat peritoneal mast cells and passive cutaneous anaphylaxis [3]. Recently, a study demonstrated the anti-allergic effect of skullcap against an egg allergen, ovalbumin (OVA), in which permeation is suppressed by skullcap via the upregulation of occludin, ZO-1, and JAM expression in the intestinal epithelial cells [4]. Although studies on the physiological functions of skullcap, such as anti-allergic effects, have progressed actively, the detailed functions and mechanisms have not been elucidated thus far.

Skullcap contains many constituents, and more than 60 structures (for example; oroxylin A, paconiflorin, glycyrrhetinic acid, liquiritigenin, isoliquiritigenin and ononin) have been identified [5]. Among these components, baicalein, baicalin, and wogonin are known to be the major flavonoids of skullcap. The anti-inflammatory and anti-cancer effects of these compounds in a disease model were reported [6,7,8,9]. However, compared with their anti-inflammatory effects, their anti-allergic effects have not been described yet. Furthermore, the anti-allergic effects of components have been studied mainly with mast cell-mediated responses or peripheral immune responses such as atopic dermatitis and asthma [10,11,12].

**Figure 1:**
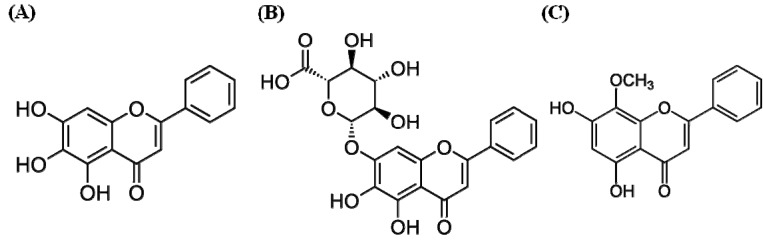
Chemical structures of the active components of skullcap: baicalein (**A**), baicalin (**B**), and wogonin (**C**).

In this study, we investigated the anti-allergic effect of skullcap extract and its known active compounds—baicalein, baicalin, and wogonin (Figure 1), with a focus on the systemic immunity T cell-mediated immune response. The effects of skullcap and its active components were evaluated by immunoglobulin (Ig) and cytokine analyses of an OVA-induced Th2 dominant mouse model.

## 2. Results

### 2.1. Effects of Skullcap Extract on the OVA-Induced Th2 Immune Response in *Ex Vivo*

**Figure 2:**
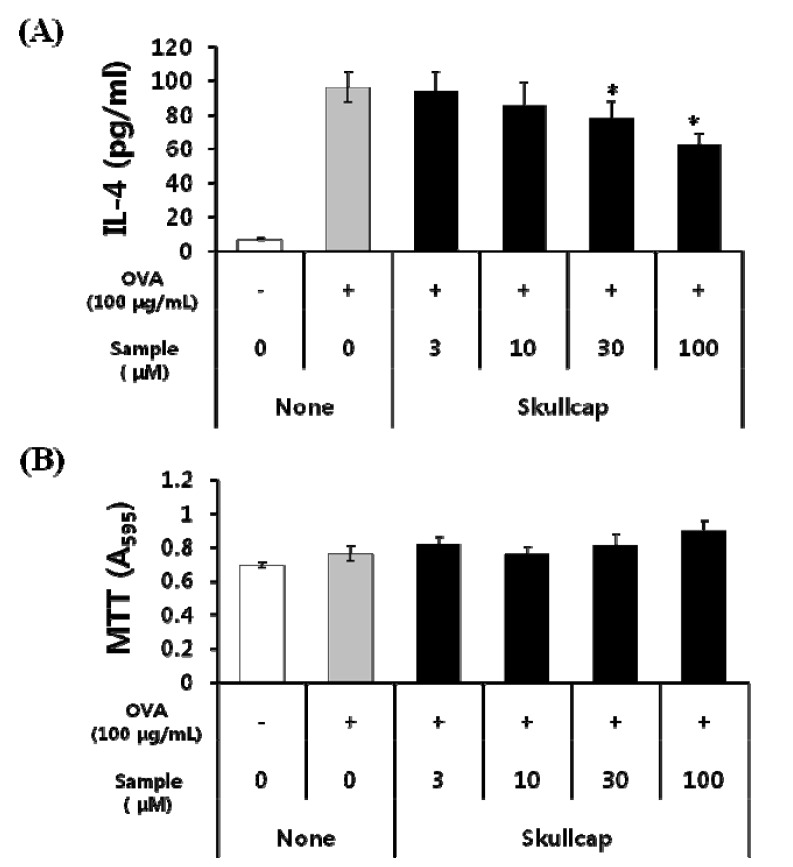
Effects of skullcap extract on OVA-induced Th2 immune response* ex vivo.*

We examined the anti-allergic effects of the skullcap extract on splenocytes isolated from OVA-sensitized mice. These mice generally have a tendency for a dominant Th2 response. Skullcap extract significantly inhibited interleukin (IL)-4 production in a dose-dependent manner (Figure 2A). We also determined cell viability to confirm whether the inhibitory effect of skullcap extract on IL-4 production was caused by cytotoxicity or cell damage. We found that viability of splenocytes was not significantly different when treated with the skullcap extract (Figure 2B). These results showed that the skullcap extract may regulate the immune response by inhibiting IL-4 production without cytotoxicity.

Splenocytes isolated from OVA-sensitized mice were incubated with both 100 µg/mL OVA and 3–100 µg/mL skullcap extract for 72 h. IL-4 production was detected by an ELISA (A). Cell viability and cytotoxicity were measured by the 3-(4, 5-dimethylthiazol-2-yl)-2, 5-diphenyltetrazolium bromide (MTT) assay (B). Each value represents the mean ± SD (*n* = 3). * *P <* 0*.*05 and ** *P <* 0*.*01* vs.* the control. Data were analyzed using ANOVA followed by F-protected Fisher’s least significant difference test.

### 2.2. Effects of Baicalein, Baicalin, and Wogonin on the OVA-Induced Th2 Dominant Response

**Figure 3:**
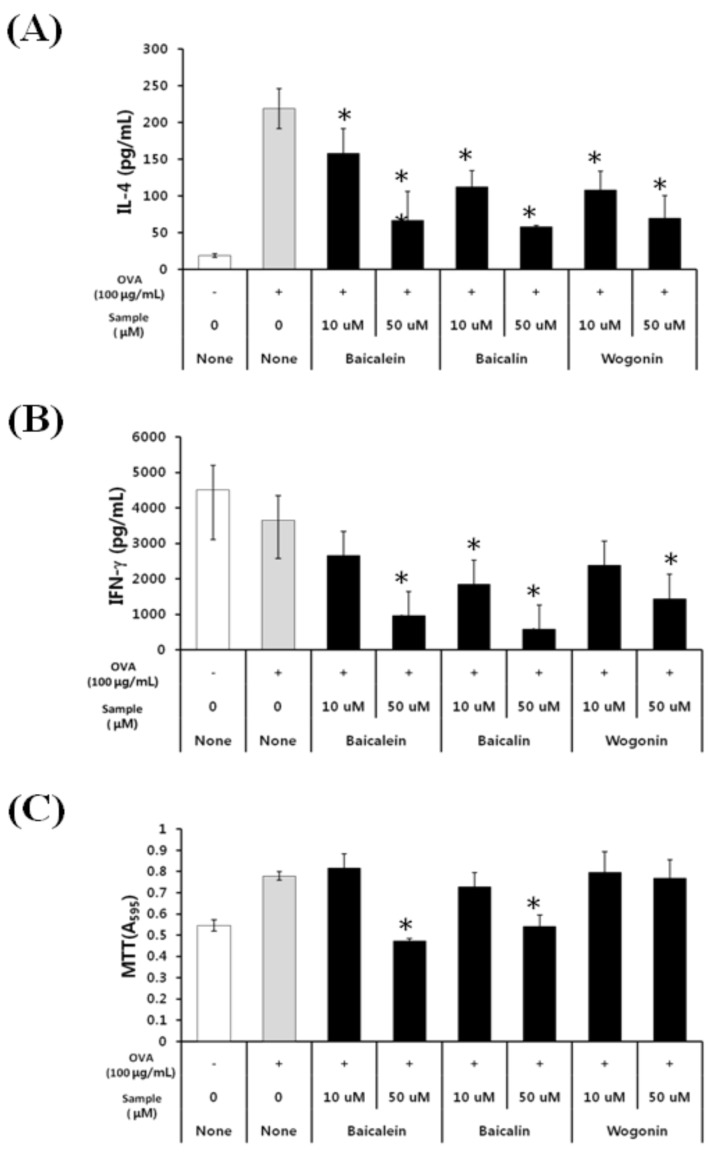
Effects of baicalein, baicalin, and wogonin on OVA-induced Th2 immune response.

We next investigated the effects of the active compounds in skullcap, including baicalein, baicalin, and wogonin, on the OVA-induced Th2 dominant immune response. We found that baicalein, baicalin, and wogonin inhibited IL-4 production in a dose-dependent manner (Figure 3A). Similarly, baicalein, baicalin, and wogonin dose-dependently decreased interferon gamma (IFN-γ) production (Figure 3B). The active components also inhibited other Th2 cytokines (IL-5, IL-13, and IL10) and other Th1 cytokine (IL-12) (data not shown). Interestingly, the viability of cells treated with wogonin did not decrease compared with that of cells treated with OVA, but cell viability was decreased by treatment with baicalein and baicalin (Figure 3C). These results indicate that wogonin mainly affects OVA-induced production of IL-4 and other cytokines, without affecting the cell viability of splenocytes.

Splenocytes isolated from OVA-sensitized mice were treated with both 100 µg/mL OVA and 10–50 µmol/L skullcap active components for 72 h. IL-4 levels and IFN-γ production were detected by ELISAs (**A,B**). Cell viability and cytotoxicity were measured by MTT assay (**C**). Each value represents the mean ± SD (*n* = 3). * *p <* 0*.*05 and ** *p <* 0*.*01* vs.* the control. Data were analyzed using ANOVA followed by F-protected Fisher’s least significant difference test.

### 2.3. Effects of Wogonin by Oral Gavage on OVA-Induced Immune Responses *In Vivo*

**Figure 4:**
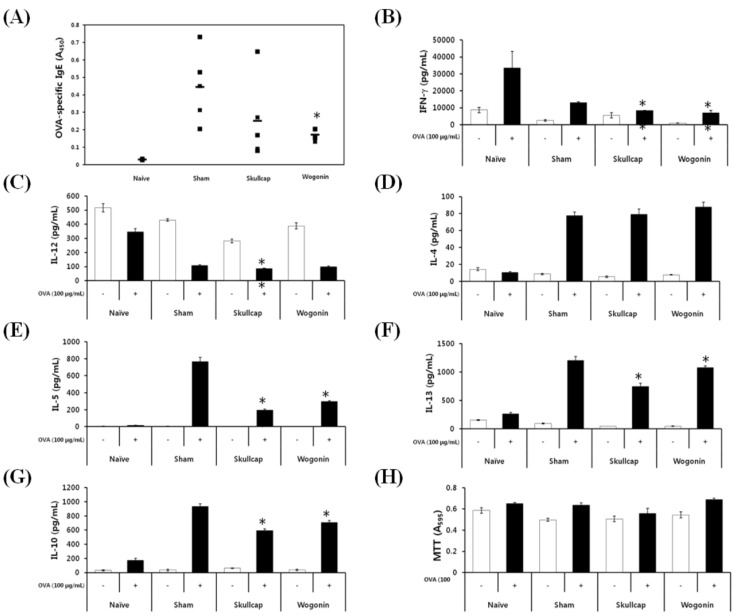
Effects of wogonin by oral gavage on OVA-induced immune responses* in vivo.*

Then, we examined the effects of wogonin and skullcap extract by oral administration *in vivo*. The animal experiment schedule is shown in Section 4.4. We investigated the amount of OVA-specific IgE, IgG2a, and IgG1 in sera by ELISA. We found that both the skullcap and wogonin significantly inhibited OVA-specific IgE production (Figure 4A). However, neither the skullcap nor wogonin inhibited the production of OVA-induced IgG1 and IgG2a (data not shown).

Mice sensitized with OVA were treated with skullcap extract at 25 mg/kg of body weight (BW) or wogonin at 1 mg/kg of BW for 16 days by oral administration. Ig levels (IgE, IgG1, and IgG2a) in the serum were analyzed (**A**), and splenocytes were analyzed for production of cytokines (IFN-γ, IL-12, IL-4, IL-5, IL-10, and IL-13) (**B–G**). Each Ig and cytokine was detected by an ELISA. And cell viability and cytotoxicity were measured by MTT assay after cell separation of splenocytes (**H**). Each value represents the mean ± SD (*n* = 5). * *p* *<* 0*.*05 and ** * p* *<* 0*.*01* vs.* the control. Data were analyzed using ANOVA followed by F-protected Fisher’s least significant difference test.

We also investigated the effects of skullcap and wogonin on the production of Th2-related cytokines (including IFN-γ and IL-12) and Th1-related cytokines (including IL-4, IL-5, IL-10, and IL-13) in splenocytes. IFN-γand IL-12 production was weakly decreased by skullcap and wogonin (Figure 4B,C). In addition, the production of IL-5, IL-10, and IL-13, but not IL-4 was significantly suppressed by treatment with skullcap and wogonin (Figure 4D–G). Furthermore, Figure 4H was shown that both skullcap extract and wogonin suppressed the cytokines without cytotoxicity. These results indicate that skullcap and wogonin inhibit the production of IgE and Th2-mediated cytokines, especially IL-5, thus resulting in suppression of Th2-mediated allergic disorders.

## 3. Discussion

Three flavonoids have been identified as the major active components of skullcap: baicalein, baicalin, and wogonin. Each of these active components is known to have physiological effects as well as cytotoxic or side effects when used at high concentrations. In this study, we examined the anti-allergic effect of baicalin, baicalein, and wogonin, and we evaluated the viability of splenocytes treated with each of these active components. Treatment with baicalein and baicalin showed a decrease in the cell viability at 50 µmol/mL (Figure 3). Inhibitory effects of baicalin and baicalein on IFN-γ and IL-4 production were assumed to also affect cell viability or induce cytotoxicity. Recently, it was reported that baicalin induces naïve CD4^+^ T cells to CD4^+^CD25^+^Foxp3^+^ T cells and suppresses Th1- and Th2-mediated immune responses via inhibition of cell proliferation [13]. Moreover, baicalein induces apoptosis in human leukemia HL-60 and Jurkat cells [14]. Our results also showed that baicalein and baicalin suppressed cell viability, which supports both regulatory T cell induction by baicalin and apoptosis induction by baicalein. However, wogonin treatment showed no effect on the cell viability, but suppressed IL-4 and IFN-γproduction, similar to the effects of skullcap treatment. Therefore, these results indicate that the active compound in skullcap, which affects the Th2-dominant allergic response by OVA, would likely be wogonin.

In the present study, our results showed that the effect of skullcap or wogonin on IL-4 production induced by OVA in* in vitro* was different to the observation in the* in vivo* experiment. We considered why the skullcap or wogonin was different on IL-4 production between* in vitro* and* in vivo*. Splenocytes were directly treated with skullcap extract or wogonin in* in vitro* experiment. It means that the skullcap or wogonin directly affected to splenocytes including APCs and T cells for inhibition of IL-4. Meanwhile, in* in vivo* experiment, the skullcap or wogonin indirectly affected to splenocyte in systemic immunity via intestinal immune systems (PP, MLN, and LP). Furthermore, the skullcap or wogonin administered by P.O. might be metabolized other forms by many factors such as emzymes and microflora. Thus, we thought that the results between* in vitro* and* in vivo* could be different on IL-4 production.

Orally administered skullcap or wogonin may suppress the production of Th1- and Th2-mediated cytokines in intestinal immune systems because they can directly affect immune cells in the intestine like in the* in vitro* experiments. However, since the systemic immune system was affected many other immune cells including T cells, APCs, eosinophils, and mast cells, Th2-dominat response induced by OVA might produce IL-5^high^ Th2 cells [15]. Because our* in vivo* results showed that OVA induced low levels of IL-4 production compared with IL-5 and IL-13. If the IL-5^high^ Th2 cells presented in splenocytes, the strong inhibitory effect of skullcap or wogonin could be explained by the* in vivo* experiment.

*In vivo*, skullcap and wogonin significantly suppressed IL-5 production, but did not inhibit IL-4 production. We considered that skullcap and wogonin might selectively act in the signaling pathway of IL-5 production in Th2 cells. Recently, it was reported that Th2 cells, especially the CD62L^l^°^w^CXCR3^l^°^w^ population, produce high amounts of IL-5 via inhibition of transcriptional factor eomesodermin. This observation indicates that eomesodermin may control IL-5 production in Th2 cells by inhibiting the activity of the transcription factor GATA3 [16]. Therefore, skullcap and wogonin might exert an influence on Th2 cells to suppress IL-5 production via regulation of IL-5-related transcriptional factors such as eomesodermin and GATA3. However, detailed mechanisms remain to be elucidated, and we intend to investigate these mechanisms in our future study.

IgE plays an essential role in type 1 hypersensitivity, and the level of IgE is significantly increased in serum or plasma in various allergic disorders such as asthma, allergic rhinitis, atopic dermatitis, and food allergy [17,18]. In this study, the IgE level in serum increased by OVA administration, and skullcap and wogonin treatment suppressed the IgE level. Our results suggest that skullcap and wogonin might directly downregulate IgE production through B cells, and indirectly reduce the IgE level via inhibition of IL-5 production. A correlation between IL-5 and IgE has been reported to increase or decrease IL-5 to regulate IgE production [19,20]. In the present study, skullcap and wogonin strongly inhibited IgE and IL-5 production induced by OVA. Based on these observations, we suggest that skullcap and wogonin might reduce the IgE level via suppression of IL-5 production.

## 4. Experimental

### 4.1. Materials

RPMI 1640 medium, fetal bovine serum, penicillin-streptomycin, and Dulbecco’s phosphate-buffered saline were purchased from WelGENE (Daegu, Korea). OVA (grade VI), MTT, and sodium dodecyl sulfate were purchased from Sigma-Aldrich (St. Louis, MO, USA). Baicalin, baicalein, and wogonin were purchased from Wako Pure Chemicals Inc., Ltd. (Osaka, Japan). Skullcap extract was provided from the Korea Food Research Institute (specimen No.; KFRI-SL-101).

### 4.2. Animals

Female BALB/c mice, weighing approximately 18–20 g, were purchased from OrientBio Inc. (Kyeonggi, Korea). Female Balb/c mice (6 weeks old) were housed in an air-conditioned room (23 °C ± 2 °C) with a 12 h light/dark cycle. All animal experiments were performed in accordance with the guidelines for animal use and care of the Korea Food Research Institute.

### 4.3. Sensitization and Challenge with OVA and Preparation of Splenocyte Cultures

**Figure 5:**
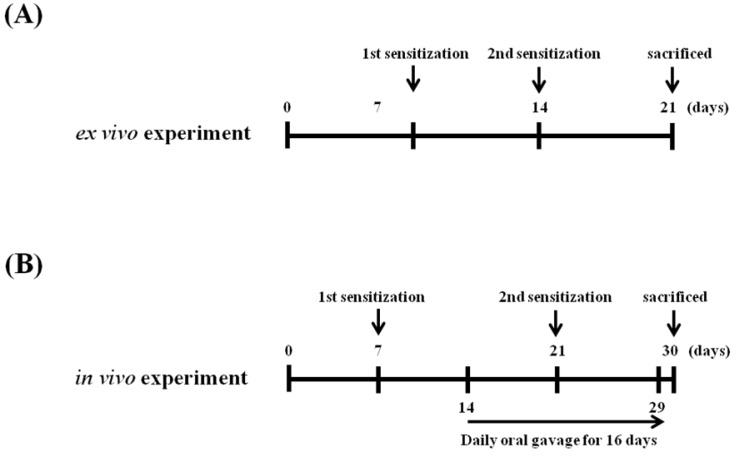
Schamatic diagram of the experiments of* ex vivo* and* in vivo.*

Mice were sensitized with 20 μg OVA adsorbed in 2 mg/mL Imject Alum (Pierce, Rockford, IL, USA) by intraperitoneal (i.p.) injection on days 7 and 14 (Figure 5A). Splenocytes were prepared by aseptically removing the spleens from OVA-sensitized BALB/c mice. The tissue was homogenized, and cells were collected and treated with red blood cell lysis buffer. The number of splenocytes was adjusted to a cell density of 5 × 10^6^ cells/mL in RPMI 1640 medium by using the trypan blue exclusion method. Splenocytes were then cultured in the absence or presence of 100 μg/mL OVA at 37 °C for up to 72 h in a humidified incubator with 5% CO_2_.

### 4.4. Schedules for Mice Sensitization, OVA Challenges, and Sample Treatment *In Vivo*

A schematic of the experimental procedure is shown in Figure 5B. Mice were divided into the following four groups: naïve, sham, skullcap, and wogonin groups (*n* = 5). Mice were sensitized with 20 μg OVA adsorbed in 2 mg/mL Imject Alum by i.p. injection on days 7 and 21. In the treatment groups, skullcap extract (25 mg/kg of BW) or wogonin (1 mg/kg of BW) was orally administered for 16 days (days 14–29). The concentration of wogonin was calculated from the extraction yield and polyphenol content. Wogonin was mainly detected in the butanol fraction, and the yield and total polyphenol content of the fraction was 40% (*w/w*) and 13.38% ± 0.07% (*w/w*), respectively. These results indicated that 1.3 mg of wogonin was present in 25 mg of skullcap. Thus, oral administration of wogonin at 1 mg/kg BW was equivalent to administration of skullcap extract at 25 mg/kg BW. And then the mice were killed on day 30. For analysis of Igs, serum samples were obtained by collecting the blood from the orbital venous plexus. Spleens were removed and were used for cytokine production analysis and MTT assays.

### 4.5. Measurement of Cytokine Levels by ELISA

ELISA kits (BD PharMingen, San Diego, CA, USA) were used for the measurement of cytokines (IFN-γ, and IL-12, IL-4, IL-5, IL-10, and IL-13) released into culture supernatants, according to the manufacturer’s instructions.

### 4.6. Cytotoxicity/Viability Assay

Cytotoxicity of splenocytes was determined by MTT assays. Briefly, the cells were seeded in 96-well plates at a density of 5 × 10^6^ cells/mL and incubated with various concentrations of the skullcap extract, baicalin, baicalein, or wogonin for 72 h. The cells were then washed twice with phosphate-buffered saline, and 20 µL of MTT solution (2 mg/mL) was added to each well. After incubation at 37 °C in a 5% CO_2_ atmosphere for 4 h, the cells were dissolved in 100 µL of 10% sodium dodecyl sulfate. The amount of formazan was determined by measuring the absorbance at 595 nm by using an Epoch microplate reader (BioTek, Winooski, VT, USA).

### 4.7. Statistical Analysis

Data are expressed as mean ± standard deviation (SD). Differences between experimental data were assessed by one-way analysis of variance (ANOVA) followed by F-protected Fisher’s least significant difference test.

## 5. Conclusions

In the present study, we demonstrated that wogonin, an active component of skullcap, downregulates OVA-induced Th2 immune responses, especially IgE and IL-5 production. Wogonin may be applied as a preventive and therapeutic agent for IgE- and IL-5-mediated allergic disorders such as food allergy, atopic dermatitis, and asthma.

## References

[1] Yoon S.B., Lee Y.J., Park S.K., Kim H.C., Bae H., Kim H.M., Ko S.G., Choi H.Y., Oh M.S., Park W. (2009). Anti-inflammatory effects of Scutellaria baicalensis water extract on LPS-activated RAW 264.7 macrophages. J. Ethnopharmacol..

[2] Kim E.H., Shim B., Kang S., Jeong G., Lee J.S., Yu Y.B., Chun M. (2009). Anti-inflammatory effects of Scutellaria baicalensis extract via suppression of immune modulators and MAP kinase signaling molecules. J. Ethnopharmacol..

[3] Jung H.S., Kim M.H., Gwak N.G., Im Y.S., Lee K.Y., Sohn Y., Choi H., Yang W.M. (2012). Antiallergic effects of Scutellaria baicalensis on inflammation *in vivo* and *in vitro*. J. Ethnopharmacol..

[4] Shin H.S., Bae M.J., Jung S.Y., Shon D.H. (2013). Inhibitory effect of skullcap (*Scutellaria. baicalensis*) extract on ovalbumin permeation *in vitro* and *in vivo*. Food Chem..

[5] Li H.B., Jiang Y., Chen F. (2004). Separation methods used for Scutellaria baicalensis active components. J. Chromatogr. B.

[6] Malbalirajan U., Ahmad T., Rehman R., Leishangthem G.D., Dinda A.K., Agrawal A., Ghosh B., Sharma S.K. (2013). Baicalein reduces airway injury in allergen and IL-13 induced airway inflammation. Separation methods used for Scutellaria baicalensis active components. PLoS One.

[7] Guo M., Zhang N., Li D., Liang D., Liu Z., Li F., Fu Y., Cao Y., Deng X., Yang Z.  (2013). Baicalin plays an anti-inflammatory role through reducing nuclear factor-κB and p38 phosphorylation in S. aureus-induced mastitis. Int. Immunopharmacol..

[8] Yeh C.H., Shih H.C., Hong H.M., Lee S.S., Yang M.L., Chen C.J., Kuan Y.H. (2013). Protective effect of wogonin on proinflammatory cytokine generation via Jak1/3-STAT1/3 pathway in lipopolysaccharide stimulated BV2 microglial cells. Toxicol. Ind. Health.

[9] Li-Weber M. (2009). New therapeutic aspects of flavones: The anticancer properties of Scutellaria and its main active constituents wogonin, baicalein and naicalin. Cancer Treat. Rev..

[10] Hsieh C.J., Hall K., Ha T., Li C., Krishnaswamy G., Chi D.S. (2007). Baicalein inhibits IL-1β-and TNF-α-induced inflammatory cytokine production from human mast cells via regulation of the NF-kappaB pathway. Clin. Mol. Allergy.

[11] Sun J., Li L., Wu J., Liu B., Gong W., Lv Y., Luo Q., Dun X., Dong J. (2013). Effects of balicalin on airway remodeling in asthmatic mice. Planta Med..

[12] Lim H., Park H., Kim H.P. (2004). Inhibition of contact dermatitis in animal models and suppression of proinflammatory gene expression by topically applied flavonoid, wogonin. Arch. Pharm. Res..

[13] Yang J., Yang X., Chu Y., Li M. (2011). Identification of Baicalin as an immunoregulatory compound by controlling T(H)17 cell differentiation. PLoS One.

[14] Chow J.M., Shen S.C., Wu C.Y., Chen Y.C. (2006). 12-o-Tetradecanoylphorbol 13-acetate prevents baicalein-induced apoptosis via activation of protein kinase C and JNKs in human leukemia cells. Apoptosis.

[15] Debes G.F., Diehl M.C. (2011). CCL8 and skin T cells—An allergic attraction. Nat. Immunol..

[16] Endo Y., Iwamura C., Kuwahara M., Suzuki A., Sugaya K., Tumes D.J., Tokoyoda K., Hosokawa H., Yamashita M., Nakayama M. (2011). Eomesodermin controls interleukin-5 production in memory T helper 2 cells through inhibition of activity of the transcription factor GATA3. Immunity.

[17] Gould H.J., Sutton B.J., Beavil A.J., McCloskey N., Coker H.A., Fear D., Smurthwaite L. (2003). The bioloy of IgE and the basis of allergic disease. Annu. Rev. Immunol..

[18] Burrow B., Martinez F.D., Halonen M., Barbee R.A., Cline M.G. (1989). Association of asthma with serum IgE levels and skin-test reactivity to allergens. N. Engl. J. Med..

[19] Crestani E., Lohman I.C., Guerra S., Wright A.L., Halonen M. (2007). Association of IL-5 cytokine production and *in vivo* IgE levels in infants and parents. J. Allergy Clin. Immun..

[20] Losol P., Kim S.H., Hwang E.K., Shin Y.S., Park H.S. (2013). IL-5 Promoter polymorphism enhances IgE responses to staphylococcal superantigens in adult asthmatics. Allergy Asthma Immunol. Res..

